# Natural Essential Oils as Promising Antimicrobial Agents to Improve Food Safety: Mechanistic Insights Against Multidrug-Resistant *Campylobacter jejuni* and *Campylobacter coli* Isolated from Tunisia

**DOI:** 10.3390/foods15020308

**Published:** 2026-01-14

**Authors:** Manel Gharbi, Chedia Aouadhi, Chadlia Hamdi, Safa Hamrouni, Abderrazak Maaroufi

**Affiliations:** 1 Group of Bacteriology and Biotechnology Development, Laboratory of Epidemiology and Veterinary Microbiology, Institut Pasteur de Tunis, University of Tunis El Manar (UTM), Tunis 1002, Tunisiachadlia.hamdi@pasteur.utm.tn (C.H.); safa.hamrouni@pasteur.tn (S.H.); abderrazak.maaroufi@pasteur.tn (A.M.); 2 Higher Institute of Biotechnology of Beja, University of Jendouba, Beja 9000, Tunisia

**Keywords:** multidrug-resistant *Campylobacter*, essential oils, natural antimicrobials, antibacterial mechanisms, food safety improvement

## Abstract

The increasing prevalence of multidrug-resistant (MDR) *Campylobacter* species poses a serious threat to food safety and public health, highlighting the urgent need for natural antimicrobial alternatives to conventional antibiotics. This study investigated the antibacterial potential and mechanism of action of seven essential oils (EOs), *Cymbopogon citratus*, *Mentha pulegium*, *Artemisia absinthium*, *Myrtus communis*, *Thymus algeriensis*, *Thymus capitatus*, and *Eucalyptus globulus*, against multidrug-resistant *Campylobacter jejuni* and *Campylobacter coli*. The antimicrobial activity was first assessed by the agar disk diffusion and broth microdilution methods to determine inhibition zones, minimum inhibitory concentrations (MICs), and minimum bactericidal concentrations (MBCs). The most active EOs were further evaluated through time–kill kinetics, cell lysis, salt tolerance, and membrane integrity assays to elucidate their bactericidal mechanisms. Results showed that *E. globulus*, *T. algeriensis*, and *M. communis* exhibited the strongest inhibitory effects, particularly against *C. jejuni*, with MIC values ranging from 3.125% to 6.25%, while *C. coli* was more resistant. Time–kill and lysis experiments demonstrated rapid bacterial reduction and significant decreases in optical density, indicating cell disruption. Additionally, EO treatments reduced salt tolerance and induced leakage of cytoplasmic materials, confirming membrane damage. Overall, these findings suggest that selected essential oils exert potent antimicrobial effects through membrane disruption and osmotic imbalance, offering promising natural strategies to control *MDR Campylobacter* in food systems. The application of such bioactive compounds could contribute significantly to improving food quality, extending shelf life, and enhancing food safety.

## 1. Introduction

*Campylobacter* species, particularly *Campylobacter jejuni* and *Campylobacter coli*, are among the leading bacterial causes of foodborne gastroenteritis worldwide, often linked to the consumption of contaminated poultry and other animal-derived products [1,2]. In humans, infections are typically characterized by diarrhea, abdominal cramps, fever, and vomiting, but in vulnerable populations such as young children, the elderly, and immunocompromised individuals, infections can lead to severe complications including bacteremia, reactive arthritis, and, in rare cases, Guillain–Barré syndrome [3,4]. The management of *Campylobacter* infections is further complicated by the rising prevalence of multidrug-resistant (MDR) strains, which exhibit resistance to commonly used antibiotics such as fluoroquinolones, macrolides, and tetracyclines. This trend has become a major global concern, as it compromises the effectiveness of conventional antibiotic therapies, increases the risk of treatment failure, and poses a significant threat not only to human health but also to food production systems, where MDR *Campylobacter* can spread through the poultry and livestock supply chain [5,6,7]. The persistence of these resistant strains in animal reservoirs highlights the urgent need for alternative intervention strategies and comprehensive surveillance programs to mitigate their impact on public health.

The intensive and sometimes uncontrolled use of antibiotics in livestock farming has promoted the selection and spread of resistant *Campylobacter* strains throughout the food chain [8,9]. This contributes to the emergence of MDR populations and increases the risk of transmission to humans through contaminated animal products. Antibiotic overuse also raises concerns about environmental contamination and the persistence of resistant bacteria in agricultural settings. These challenges emphasize the pressing need for alternative approaches to control pathogenic bacteria in a manner that does not exacerbate antibiotic resistance. Plant-derived essential oils (EOs) are among the most promising options, as they exhibit broad-spectrum antimicrobial activity and are naturally biodegradable. Several EO constituents have been recognized as Generally Recognized as Safe (GRAS) by regulatory authorities; however, this designation is context-dependent and varies according to the specific compound, concentration, intended application, food matrix, and regulatory jurisdiction. In addition, sensory attributes and consumer acceptance may constrain their practical use. Despite these limitations, EOs offer considerable potential for application in food systems and animal production to reduce microbial loads, limit pathogen dissemination, and mitigate the risks associated with antibiotic overuse [10,11].

EOs are complex mixtures of volatile compounds such as terpenes, phenols, and ketones that often interact with bacterial membranes, increase permeability, and cause leakage of intracellular contents, ultimately leading to cell death [12,13]. Recent studies have demonstrated significant antibacterial activity of various essential oils against *Campylobacter* spp. For instance, formulations containing cinnamon, lemongrass, clove, geranium, and oregano EOs have shown strong inhibitory effects on *Campylobacter* strains isolated from chicken carcasses, with large inhibition zones in diffusion assays and low effective concentrations when incorporated into lipid nanocarriers [14,15,16]. Moreover, dietary supplementation with natural phenolic compounds such as eugenol and trans-cinnamaldehyde has been shown to reduce *C. jejuni* loads in broiler chickens and modulate virulence gene expression, demonstrating the in vivo potential of these natural antimicrobials [17].

Despite these promising findings, the precise antibacterial mechanisms of EOs against MDR *Campylobacter* strains remain incompletely understood. The complex mixture of bioactive compounds in EOs can act through multiple pathways, but the specific interactions that lead to bacterial inhibition or cell death are not fully characterized. Understanding these mechanisms is critical for determining optimal concentrations, delivery methods, and combinations with other antimicrobials to maximize their effectiveness. Insights into the bactericidal action of EOs will also facilitate translation of laboratory efficacy into practical applications in food production systems, ensuring both safety and consistency of antimicrobial effects [18,19].

Therefore, the present study aimed to evaluate the antibacterial activity of seven essential oils: *Cymbopogon citratus*, *Mentha pulegium*, *Artemisia absinthium*, *Myrtus communis*, *Thymus algeriensis*, *Thymus capitatus*, and *Eucalyptus globulus* against MDR *C. jejuni* and *C. coli* strains, and (ii) elucidate their mechanisms of action through time–-kill kinetics, cell lysis, salt tolerance, and membrane integrity assays. The findings of this work provide new insights into the bactericidal properties of EOs and support their application as natural antimicrobial agents to enhance food safety and reduce antibiotic dependence in poultry production.

## 2. Materials and Methods

### 2.1. Essential Oils

In the present study, seven commercially obtained EOs, *Cymbopogon citratus*, *Mentha pulegium*, *Artemisia absinthium*, *Myrtus communis*, *Thymus algeriensis*, *Thymus capitatus*, and *Eucalyptus globules*, were purchased from the company “Carthago Essences Sousse, Tunisia”, and evaluated for their antibacterial activity against MDR *C. jejuni* and *C. coli* strains isolated from poultry in Tunisia. The oils were supplied with a purity ≥ 98% according to the manufacturer’s specifications. Detailed information on the composition and concentration of specific active compounds was not provided by the supplier.

### 2.2. Selected Bacteria and Growth Conditions

Ten multidrug-resistant *Campylobacter* strains (Table 1), including five *C. jejuni* and five *C. coli* isolates, were used to evaluate the antibacterial activity of the essential oils. The strains were obtained from poultry sources. Their antibiotic susceptibility profiles had been previously determined by [20,21]. All strains were cultured on Karmali agar (Oxoid, Basingstoke, UK) and incubated under microaerophilic conditions (5% O_2_, 10% CO_2_, 85% N_2_) at 42 °C for 48 h.

### 2.3. Preliminary Assessment of the Antimicrobial Activity of Essential Oils

The antibacterial activity of the seven EOs was initially screened using the disc diffusion method, as described by Aouadhi et al. [22], with minor modifications. Briefly, Mueller-Hinton agar (Oxoid, Ltd., Basingstoke, UK) plates (without any supplementation) were inoculated by uniformly surface spreading 100 µL of each strain suspension standardized to 0.5 McFarland (10^8^ CFU/mL; OD_600_ = 0.08–0.1). Sterile filter paper discs (6 mm diameter) were impregnated with 15 µL of each EO and placed onto the inoculated agar surface. To reduce EO volatilization, plates were left at room temperature for 30 min to allow pre-diffusion before incubation. The plates were incubated in sealed bags filled with a microaerophilic gas mixture (5% O_2_, 10% CO_2_, and 85% N_2_) for 18 to 19 h at 42 °C. The diameter of the growth inhibition zone (including disc diameter of 6 mm) was used to estimate the qualitative antimicrobial activity of tested EOs. Erythromycin (15 µg/disc) was used as a positive reference. Negative control corresponds to disc without sample.

### 2.4. Quantitative Evaluation of the Antibacterial Efficacy of Essential Oils

The quantitative antibacterial activity of the seven EOs was assessed by determining their minimum inhibitory concentrations (MICs) and minimum bactericidal concentrations (MBCs). The broth microdilution method described by Aouadhi et al. [22] was employed. Bacterial suspensions were prepared by inoculating Bolton broth with three to four freshly isolated colonies, followed by incubation at 42 °C for 24 h. EOs were dissolved in dimethyl sulfoxide (DMSO) (Biomatik, ON, Canada).to achieve final concentrations ranging from 0.07% to 50% (*v*/*v*). Cultures without EOs served as positive growth controls, while negative controls contained the test EO but no bacteria. After 24 h of incubation at 42 °C, the MIC was defined as the lowest EO concentration that completely inhibited visible bacterial growth. To determine MBCs, 100 µL aliquots from each well were plated onto Karmali agar and incubated at 42 °C to evaluate cell viability. All experiments were conducted in triplicate to ensure reliability and reproducibility of the results.

### 2.5. Investigation of the Mechanism of Action of Thymus Capitatus Essential Oil

#### 2.5.1. Time–Kill Kinetics Assay

The bactericidal kinetics of *T. algeriensis* and *E. globulus* EOs were evaluated against only the five *C. jejuni*, selected based on their higher susceptibility to these oils, in order to characterize their time-dependent antibacterial activity. Each strain was tested separately and displayed a similar kinetic trend; therefore, the results are presented as the mean response of the five strains. This assay quantifies the reduction in viable bacterial counts in the presence of each EO at its minimum inhibitory concentration (MIC) [23]. Briefly, three to five colonies from an 18 h culture were suspended in 2 mL of peptone water (Oxoid, Ltd., Basingstoke, UK) to obtain a working suspension adjusted to 10^5^ CFU/mL (OD_600_ = 0.01). The EOs were added at their respective MICs, and bacterial suspensions with or without EO (growth control) were incubated at 42 °C under continuous agitation for 24 h. Aliquots (100 µL) were collected at predetermined time points (0, 2, 4, 6, 8, and 24 h) to determine bacterial survival [24].

#### 2.5.2. Bacteriolytic Activity Assay

The potential bacteriolytic effect of *T. algeriensis* and *E. globulus* EO was evaluated by monitoring the optical density of bacterial suspensions at 620 nm over time [23,24]. Two colonies from an 18 h culture of the five *C. jejuni* strains were inoculated into 9 mL of nutrient broth and incubated at 42 °C for 18 h under shaking conditions. Cells were harvested by centrifugation (10,000 rpm, 12 min, 4 °C), washed twice with phosphate-buffered saline (PBS) (Biomatik, ON, Canada), and resuspended in PBS containing Tween 80 (0.01%, *v*/*v*). The resulting suspension was standardized to 10^10^ CFU/mL (OD_600_ = 1.0) and divided into two tubes: one containing EO at its MIC (treated) and another without EO (untreated control). Suspensions were stirred for 120 min, homogenized, diluted (1:100), and their absorbance was immediately measured at 620 nm. Results were expressed as the percentage ratio of OD_620_ at each time point relative to that at 0 min.

#### 2.5.3. Determination of Cytoplasmic Material Leakage

The release of intracellular materials absorbing at 280 nm from the five *C. jejuni* strains cells following exposure to EOs of *T. algeriensis*, *E. globulus*, and *M. communis* was quantified to evaluate loss of membrane integrity. The assay was performed according to the method described by Carson et al. [25].

#### 2.5.4. Evaluation of Salt Tolerance Impairment

The impact of the EOs of *T. algeriensis*, *E. globulus*, and *M. communis* on the salt tolerance of the five *C. jejuni* strains was assessed according to the method described by Carson et al. [22]. Bacterial suspensions were treated or left untreated with the respective EOs at their MICs for 30 min, then plated onto nutrient agar (Oxoid, Ltd., Basingstoke, UK) supplemented with increasing concentrations of NaCl (0–100 g/L). After incubation at 42 °C for 24 h, colony-forming units (CFU/mL) were enumerated. Salt tolerance was determined by comparing CFU counts obtained on NaCl-supplemented media with those on NaCl-free agar.

### 2.6. Statistical Analysis

All experiments were conducted in triplicate and results are expressed as mean ± standard deviation (SD). Data normality was assessed using the Shapiro–Wilk test [26] and homogeneity of variances was verified using Levene’s test [27]. One-way analysis of variance (ANOVA) followed by [28]. Tukey’s honestly significant difference (HSD) post hoc test [28] was used to compare inhibition zone diameters, MIC and MBC values, and NaCl tolerance among essential oils. Two-way ANOVA was applied to assess the effects of EO type and bacterial strain, as well as their interaction, on inhibition diameters and growth reduction. Differences were considered statistically significant at *p* < 0.05. All statistical analyses were performed using GraphPad Prism version 9.5.1 (GraphPad Software, San Diego, CA, USA) and IBM SPSS Statistics version 26.0 (IBM Corp., Armonk, NY, USA).

## 3. Results and Discussion

### 3.1. Antibacterial Activity of Essential Oils (Disk Diffusion Assay)

The disk diffusion assay revealed notable variability in the antibacterial activity of the seven essential oils (EOs) tested against *C. coli* and *C. jejuni*, highlighting both strain-specific and oil-specific effects (Table 2). Two-way ANOVA confirmed that the type of EO type (*p* < 0.001) and the bacterial strain (*p* < 0.01) significantly influenced inhibition diameters, with a significant interaction between the two factors (*p* < 0.05), indicating that the effectiveness of a given EO depends on the target strain. Among the oils tested, *A. absinthium* EO showed the most potent activity (*p* < 0.05) against *C. coli*, producing the largest inhibition zone (37.6 ± 0.6 mm). In contrast, *C. citratus* and *M. pulegium* EOs displayed moderate activity against *C. coli* (28.6 ± 0.3 and 31 ± 0.2 mm, respectively) but were almost inactive against *C. jejuni*. Interestingly, the EO of *T. algeriensis* demonstrated comparable inhibitory effects against both *C. coli* and *C. jejuni*, indicating a broad antibacterial spectrum. This property could be advantageous for the development of EO-based interventions intended to control multiple *Campylobacter* strains in food production or clinical settings. Similarly, *E. globulus* showed strong activity against *C. jejuni* but lower efficacy against *C. coli*.

These variations in EO activity are consistent with the concept that the chemical composition of EOs, typically containing 20–60 bioactive constituents, plays a central role in their antimicrobial effects. The synergistic or additive interactions among major components such as thymol, carvacrol, and linalool, along with minor constituents, can significantly influence bacterial susceptibility [23,29,30]. Mechanistically, these compounds are reported to disrupt bacterial membranes, increase permeability, and induce leakage of intracellular contents, ultimately leading to cell death [31,32].

### 3.2. Minimum Inhibitory and Bactericidal Concentrations (MIC and MBC)

The broth microdilution assay provided a quantitative assessment of the antibacterial efficacy of the EOs against *C. jejuni* and *C. coli*. The MIC and MBC values revealed marked differences in potency among the oils and between the two *Campylobacter* species (*p* < 0.05) (Table 3). Notably, *E. globulus* EO exhibited the strongest inhibitory and bactericidal activity against *C. jejuni*, with MIC and MBC values of 3.13% and 1.56% *v*/*v*, respectively. *T. algeriensis* and *M. communis* also demonstrated strong activity, with MIC values of 6.25% *v*/*v* against *C. jejuni*, highlighting their potential as natural antimicrobials. In contrast, *T. capitatus* showed the weakest activity, with MICs of 25% and 50% *v*/*v* against *C. jejuni* and *C. coli*, respectively. These differences may reflect variations in EO chemical composition, particularly in the concentration and types of phenolic and monoterpene constituents, which are known to influence membrane permeability, cytoplasmic leakage, and disruption of metabolic processes in bacteria [1,2,7,29].

Interestingly, *C. coli* consistently exhibited higher MIC and MBC values compared to *C. jejuni* across most EOs (*p* < 0.01, two-way ANOVA; Table 3), indicating greater intrinsic tolerance. This trend may be related to differences in cell membrane composition, efflux pump activity, or stress response mechanisms, which could reduce the susceptibility of *C. coli* to EO components. The higher resistance of *C. coli* underscores the importance of considering species-specific responses when designing EO-based antimicrobial interventions. The observed patterns align with previous studies, including Ozogul et al. [33], which reported differential susceptibility of *Campylobacter* strains to *Thymus* spp. and *Eucalyptus* oils. The strong antimicrobial activity of these oils is generally attributed to their bioactive terpenes, including thymol, carvacrol, and 1,8-cineole, which act synergistically to destabilize the bacterial cytoplasmic membrane, disrupt membrane potential, and induce leakage of intracellular contents [23,34,35]. These mechanisms are consistent with the results of both disk diffusion and microdilution assays, reinforcing the potential of these EOs as effective natural agents against *Campylobacter* spp. Overall, the quantitative data from MIC and MBC assays confirm the species- and oil-dependent variability in antimicrobial potency and highlight *E. globulus*, *T. algeriensis*, and *M. communis* as promising candidates for further investigation.

### 3.3. Time–Kill Kinetics

**Figure 1:**
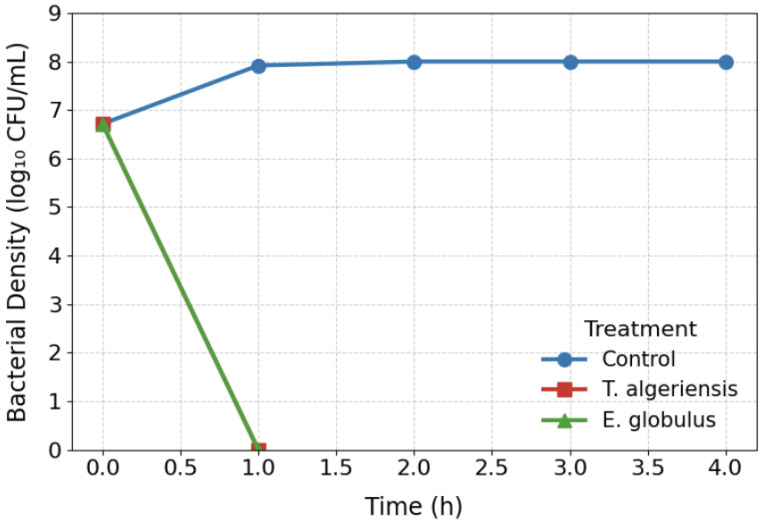
Time–kill kinetics of *T. algeriensis* and *E. globulus* essential oils against *C. jejuni*. Curves represent mean viable counts (log CFU/mL) ± SD of five independently tested *C. jejuni* strains exposed to each essential oil at its MIC.

The time–kill kinetics of *T. algeriensis* and *E. globulus* EOs against five *C. jejuni* strains were assessed to evaluate their bactericidal activity. Data shown in Figure 1 represent the mean viable counts (log CFU/mL) ± SD of the five strains, each tested individually, which all exhibited similar trends. Both EOs achieved complete inhibition of bacterial growth by the first sampling point at 1 h, indicating rapid bactericidal effects. Due to the sampling interval, it is not possible to determine whether one oil acted faster than the other; nonetheless, these results demonstrate that both oils are highly effective in rapidly reducing *C. jejuni* populations upon exposure. The rapid onset of killing suggests that the primary mechanism is likely membrane disruption rather than interference with slower biosynthetic processes, such as protein or nucleic acid synthesis. This interpretation is supported by previous studies demonstrating that monoterpenes and phenolic compounds, commonly present in these EOs, can integrate into lipid bilayers, alter membrane fluidity, induce depolarization, and cause leakage of intracellular contents, leading to cell death within minutes [31,36].

The rapid bactericidal activity observed here has several important implications. First, it supports the potential use of these EOs as effective interventions in food safety, where rapid reduction in *C. jejuni* populations is critical to prevent contamination and infection. Second, the speed of action reduces the likelihood of bacterial adaptation or development of resistance, which is a major concern with conventional antibiotics. Moreover, the differences in killing kinetics between *E. globulus* and *T. algeriensis* may reflect variations in their chemical profiles, with specific bioactive constituents such as 1,8-cineole, α-pinene, linalool, or carvacrol contributing to the observed potency and rapid membrane-targeted activity.

Interestingly, the bactericidal kinetics of these EOs resemble those reported for other plant-derived antimicrobials. For instance, Daucus carota EO was previously shown to eliminate *Campylobacter* populations within 30 min, further supporting the idea that essential oils can act as rapid, membrane-active bactericidal agents [31,36,37]. These results highlight the importance of selecting oils not only based on MIC values but also considering kinetic parameters, which provide a more comprehensive understanding of their antimicrobial potential under dynamic conditions.

### 3.4. Cell Lysis Assay

**Figure 2:**
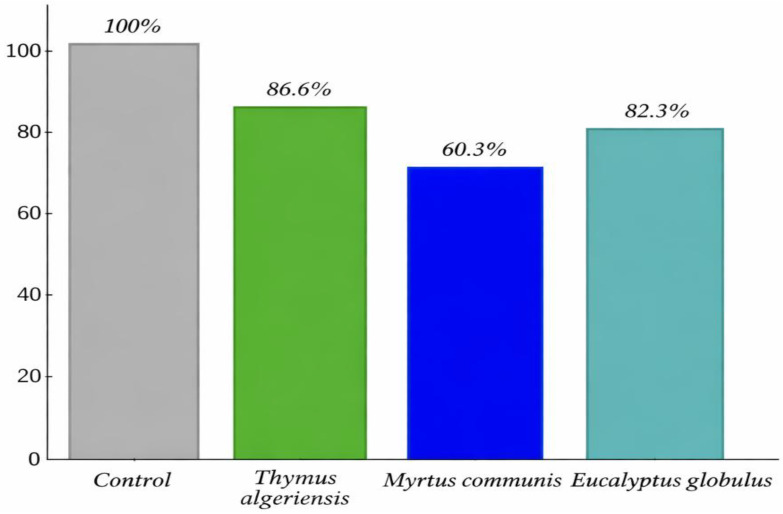
Percentage of cell lysis of five *C. jejuni* strains following treatment with selected essential oils, expressed as (A_620_(T)/A_620_(T_0_)) × 100. Values represent the mean of the five strains.

The cell lysis assay provided quantitative evidence of membrane damage and disruption of bacterial cell integrity induced by the essential oils (EOs) of *T. algeriensis*, *E. globulus*, and *M. communis*. All five *C. jejuni* strains were tested separately, and similar trends were observed across strains. Data are presented as the mean values of these five strains. A time-dependent decrease in optical density (OD_620_) was observed in all EO-treated samples, indicating progressive lysis of *C. jejuni* cells. Statistical analysis confirmed that OD_620_ values at each time point were significantly lower than the untreated control (*p* < 0.05; one-way ANOVA followed by Tukey’s post hoc test) (Figure 2). Specifically, OD_620_ decreased to 86.6%, 82.9%, and 69% of the initial absorbance for *T. algeriensis*, *E. globulus*, and *M. communis*, respectively, while the control remained stable, confirming that these effects were EO-mediated rather than due to spontaneous cell death or aggregation [30,38,39].

These results suggest that the primary antibacterial action of the tested EOs involves disruption of the bacterial cell envelope, resulting in leakage of intracellular contents and loss of turgor. The greater reduction in OD observed for *M. communis* indicates a more pronounced lytic effect compared to the other oils, likely reflecting differences in chemical composition, particularly the presence of membrane-active compounds such as phenolic monoterpenes (thymol, carvacrol) and oxygenated terpenes (1,8-cineole, linalool). These compounds are known to intercalate into phospholipid bilayers, perturb membrane structure, and destabilize lipid–protein interactions, ultimately causing cell rupture and lysis [10,11,12,13,30,35,36,38,39].

The relative differences in lytic activity among the oils are consistent with the MIC, MBC, and time–kill kinetics data, supporting a mechanism of rapid, membrane-targeted bactericidal action. For instance, *E. globules* EOs, which exhibited the lowest MIC and fastest killing kinetics, also induced substantial cell lysis, confirming that its antimicrobial potency is closely linked to membrane disruption. Similarly, the slightly lower lysis observed for *T. algeriensis* may indicate a strong but more gradual membrane-targeted effect, while *M. communis*’s pronounced OD decrease suggests an aggressive lytic action, possibly due to synergistic effects among its multiple bioactive components.

### 3.5. Effect on Salt Tolerance

The evaluation of *C. jejuni* growth on Karmali agar containing increasing NaCl concentrations (Table 4) revealed that essential oils (EOs) significantly affected bacterial tolerance to osmotic stress. Two-way ANOVA confirmed that both NaCl concentration (*p* < 0.001) and EO treatment (*p* < 0.001) had a significant impact, with a notable interaction between the two factors (*p* < 0.05). As expected, higher salt concentrations alone reduced bacterial growth, reflecting the inherent sensitivity of *C. jejuni* to osmotic stress. Interestingly, the presence of EOs amplified this effect, with *E. globulus* EOs completely inhibiting growth even at 2.5% NaCl, whereas *M. communis* and *T. algeriensis* allowed partial survival under the same conditions. These findings suggest that EO treatment interferes with the bacteria’s ability to adjust to salty conditions, likely by impairing the mechanisms responsible for maintaining ion balance and osmotic homeostasis. Disruption of osmotic regulation can prevent the cell from accumulating compatible solutes or adjusting internal solute concentrations, which is critical for survival under hyperosmotic conditions.

The differential effects of the three EOs indicate that specific chemical constituents may influence how *C. jejuni* responds to osmotic pressure. For example, *E. globulus* may contain components that either destabilize membrane-associated ion channels or inhibit stress response pathways, thereby enhancing sensitivity to NaCl. In contrast, the partial growth observed with *M. communis* and *T. algeriensis* suggests that these oils exert milder interference with osmotic adaptation mechanisms, allowing some cells to survive low to moderate salt concentrations.

These results provide additional insight into the antibacterial potential of EOs by demonstrating that, beyond direct growth inhibition, they compromise bacterial stress tolerance, which could reduce survival in challenging environments such as food matrices with high salt content. This effect could be particularly valuable for food preservation strategies, where combining EOs with mild osmotic stress may synergistically limit *C. jejuni* survival and proliferation.

Overall, the salt tolerance assay highlights a secondary mode of action for EOs: interference with bacterial adaptive responses to environmental stress, complementing their direct bactericidal activity. This dual effect reinforces the potential of EOs as multifunctional antimicrobials capable of reducing pathogen survival under diverse conditions [32,39].

### 3.6. Membrane Integrity

**Figure 3:**
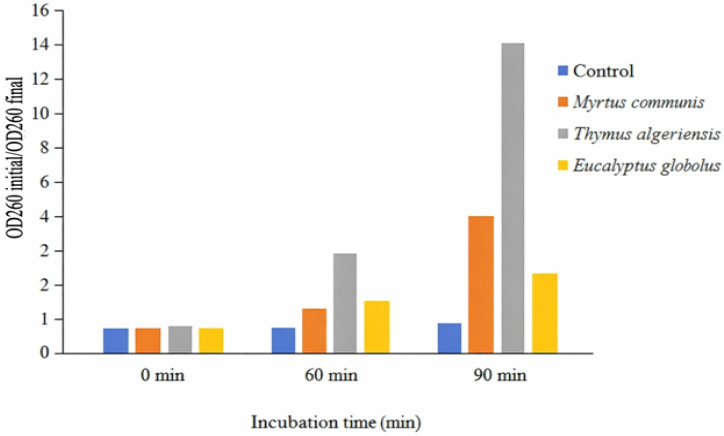
Effect of *T. algeriensis*, *E. globulus*, and *M. communis* essential oils on the release of cytoplasmic components from *C. jejuni*, measured at 260 nm. Values represent mean ± SD of five independently tested *C. jejuni* strains (*n* = 5). Statistical differences were determined using two-way ANOVA followed by Tukey’s post hoc test (*p* < 0.05).

The measurement of cytoplasmic material leakage at 260 nm provided direct evidence that *T. algeriensis*, *E. globulus*, and *M. communis* essential oils (EOs) compromise *C. jejuni* cell membrane integrity. The significant increase in OD_620_ values after EO treatment (*p* < 0.05) (Figure 3), particularly with *T. algeriensis* (OD_260_ = 14.7 after 90 min), indicates the release of nucleic acids and other intracellular components into the surrounding medium. This release is a feature of severe membrane disruption, confirming that the bacterial envelope is a primary target of EO action. The differences in leakage among the oils likely reflect variations in their chemical composition and the presence of highly active compounds such as thymol, carvacrol, and 1,8-cineole, which are known to integrate into lipid bilayers, destabilize membranes, and increase permeability [10,11,12,13,35]. *T. algeriensis* caused the greatest leakage, suggesting a particularly strong membrane-targeted effect, while *E. globulus* and *M. communis* were slightly less aggressive but still highly effective. These observations align with previous reports showing EO-induced leakage of cytoplasmic contents and oxidative stress in bacterial cells [14,38,39].

When integrated with the broader dataset, the cytoplasmic leakage results corroborate other indicators of membrane-targeted bactericidal activity. The low MIC and MBC values, rapid killing kinetics, decreased NaCl tolerance, and OD_620_-based lysis all point to the cell membrane as the primary site of EO action. This multi-parameter evidence reinforces the conclusion that *C. jejuni* cells are killed not simply by metabolic inhibition but through physical compromise of membrane integrity, resulting in uncontrolled leakage of essential intracellular materials and cell death.

The observed strain-dependent differences, with *C. coli* showing higher MIC values, suggest that membrane composition or associated stress-response mechanisms may confer relative tolerance to EO-induced membrane damage. Nonetheless, the consistent effectiveness of *E. globulus*, *T. algeriensis*, and *M. communis* across multiple assays highlights their strong and reproducible antibacterial activity, supporting their potential use as natural antimicrobials in food safety applications.

In general, the cytoplasmic material leakage assay provides direct mechanistic evidence that complements inhibition, MIC/MBC, time–kill, and salt tolerance results. Together, these findings demonstrate that selected EOs exert rapid, potent, and membrane-targeted bactericidal effects against *C. jejuni*, confirming their suitability as alternative interventions to control foodborne pathogens and reduce the risk of antibiotic-resistant *Campylobacter* strains.

## 4. Conclusions

This study demonstrates that selected EOs possess significant antibacterial activity against MDR *C. jejuni* and *C. coli* strains isolated from poultry in Tunisia. Among the seven EOs evaluated, those of *E. globulus*, *T. algeriensis*, and *M. communis* consistently exhibited the strongest inhibitory and bactericidal effects, as evidenced by large inhibition zones, low MIC and MBC values, and rapid killing kinetics. The other EOs, *T. capitatus*, A. *absinthium*, and *M. pulegium*, also demonstrated measurable antibacterial activity, although to a lesser extent, highlighting variability in EO potency and species-specific susceptibility. Notably, *C. coli* generally showed higher tolerance than C. jejuni, emphasizing the need to consider species-dependent differences when designing EO-based interventions

Mechanistic investigations revealed that the antibacterial action of these EOs is primarily membrane-targeted. The rapid bactericidal effects observed in time–kill assays, together with significant cell lysis, cytoplasmic material leakage, and impaired salt tolerance, indicate severe disruption of cell envelope integrity and loss of osmotic homeostasis. These complementary findings provide strong, multi-level evidence that EO constituents act predominantly through physical damage to bacterial membranes rather than solely through metabolic inhibition.

Importantly, the ability of EOs to compromise stress adaptation mechanisms, such as tolerance to elevated NaCl concentrations, suggests an additional mode of action that could reduce *Campylobacter* survival in food-related environments. This multifunctional activity strengthens their potential value as natural antimicrobials, particularly in food safety applications where rapid pathogen control and mitigation of antibiotic resistance risks are critical.

Nevertheless, it should be acknowledged that the EO concentrations required for antimicrobial efficacy may exceed sensory acceptance thresholds in certain food matrices, potentially limiting their direct application due to strong organoleptic effects. To address this limitation, future strategies should explore delivery systems such as micro- or nano-encapsulation, incorporation into edible coatings or active packaging, and integration within hurdle technology approaches, which may enable effective microbial control at reduced, sensory-acceptable concentrations.

Overall, the present work supports the use of *E. globulus*, *T. algeriensis*, and *M. communis* EOs as promising alternative or complementary agents for controlling antibiotic-resistant *Campylobacter* spp. in poultry production and food systems. Future studies should also focus on chemical characterization of active components, evaluation of synergistic effects with conventional preservation methods, assessment of sensory impact, and validation under real food matrix conditions to facilitate practical application.

## Figures and Tables

**Table 1:** Characteristics of used *C. jejuni* and *C. coli* strains [20,21].

Strains	Species	Phenotype of Resistance	Resistance Genes Detected
I208E17P48	*C. jejuni*	Amp, Amc, Cip, Chl, Nal, Ery, Tet, Chl, Lin	*tet(O)*, *cmeB*, *bla*_OXA-61_, *23S rRNA*, *gyrA*, *fexB*, *optrA*, *aac(6′)-Ib-cr*, *bla*_VIM_, *bla*_OXA-48_, *NDM*
I201E17P48	*C. jejuni*	Amp, Amc, Cip, Chl, Nal, Ery, Tet, Chl, Lin	*tet(O)*, *cmeB*, *bla*_OXA-61_, *23S rRNA*, *gyrA*, *bla*_VIM_, *aac(6′)-Ib-cr*, *bla*_OXA-48_
I245E17P48	*C. jejuni*	Amp, Amc, Cip, Chl, Nal, Ery, Tet, Chl, Lin	*tet(O)*, *cmeB*, *bl*_aOXA-61_, *23SrRNA*, *gyrA*, *bla*_VIM_, *aac(6′)-Ib-cr*, *bla*_OXA-48_
I255E17P48	*C. jejuni*	Amp, Amc, Cip, Chl, Nal, Ery, Tet, Chl, Lin	*tet(O)*, *cmeB*, *bla*_OXA-61_, *23SrRNA*, *gyrA*, *bla*_VIM_, *aac(6′)-Ib-cr*, *bla*_NDM_
I260E17P48	*C. jejuni*	Amp, Amc, Cip, Chl, Nal, Ery, Tet, Chl, Lin	*tet(O)*, *cmeB*, *bla*_OXA-61_, *23SrRNA*, *gyrA*, *bla*_VIM_, *aac(6′)-Ib-cr*, *bla*_NDM_
I1E17P1	*C. coli*	Amp, Cip, Chl, Ery, Tet, Chl, Gen	*tet(O)*, *cmeB*, *bla*_OXA-61_, *23SrRNA*, *gyrA*, *bla*_VIM_, *aac(6′)-Ib*, *bla*_NDM_
I267E30D10	*C. coli*	Amc, Cip, Chl, Nal, Ery, Tet, Chl, Lin	*tet(O)*, *cmeB*, *bla*_OXA-61_, *23S rRNA*, *gyrA*, *bla*_VIM_, *aac(6′)-Ib-cr*, *bla*_OXA-48_, *bla*_NDM_
I234E30A12	*C. coli*	Amp, Amc, Cip, Chl, Nal, Ery, Tet, Chl, Lin	*tet(O)*, *cmeB*, *bla_OXA-61_*, *23S rRNA*, *gyrA*, *bla*_VIM_, *aac(6′)-Ib*, *bla*_NDM_
I179E17P19	*C. coli*	Amp, Amc, Cip, Chl, Ery, Tet, Chl, Lin	*tet(O)*, *cmeB*, *bla*_OXA-61_, *23S* rRNA, *gyrA*, *bla*_VIM_, *aac(6′)-Ib*, *bla*_NDM_
I292E21P2	*C. coli*	Amp, Amc, Cip, Chl, Nal, Ery, Tet, Chl, Lin	*tet(O)*, *cmeB*, *bla*_OXA-61_, *23S* rRNA, *gyrA*, *bla*_VIM_, *aac(6*′)-*Ib*-*cr*, *bla*_NDM_

**Table 2:** Inhibition zone diameters (mm) of seven EOs against *C. coli* and *C. jejuni* strains.

EOs	*C. jejuni*	*C. coli*
*C. citratus*	6.0 ± 0.0 ^d^	28.6 ± 0.3 ^b^
*M. pulegium*	6.0 ± 0.0 ^d^	31.0 ± 0.2 ^b^
*A. absinthium*	18.3 ± 0.6 ^c^	37.6 ± 0.6 ^a^
*M. communis*	25.0 ± 0.5 ^b^	29.6 ± 0.3 ^b^
*T. algeriensis*	29.3 ± 0.3 ^b^	29.3 ± 0.5 ^b^
*T. capitatus*	20.3 ± 0.7 ^c^	31.3 ± 0.4 ^b^
*E. globulus*	34.6 ± 0.4 ^a^	23.3 ± 0.6 ^c^
*ERY*	6.0 ± 0.0 ^d^	6.0 ± 0.0 ^d^

Values represent mean ± SD (*n* = 3). Within each column, values with different superscript letters are significantly different (*p* < 0.05; one-way ANOVA followed by Tukey’s HSD test). ERY: erythromycin (control).

**Table 3:** Minimum inhibitory concentrations (MIC) and minimum bactericidal concentrations (MBC) of essential oils against *Campylobacter* spp. (% *v*/*v*).

Tested *Campylobacter* Species	*Essential oils*					
	*E. globulus*	*T. capitatus*	*T. algeriensis*	*M. communis*	*A. absinthium*	*M. pulegium*
**MIC (% *v*/*v*)**						
***C. jejuni***	3.13 ± 0.0 ^b^	25.0 ± 0.0 ^a^	6.25 ± 0.0 ^b^	6.25 ± 0.0 ^b^	25.0 ± 0.0 ^a^	–
***C. coli***	12.5 ± 0.0 ^a^	50.0 ± 0.0 ^a^	25.0 ± 0.0 ^a^	25.0 ± 0.0 ^a^	–	12.5 ± 0.0 ^a^
**MBC (% *v*/*v*)**						
***C. jejuni***	1.56 ± 0.0 ^b^	12.5 ± 0.0 ^a^	3.13 ± 0.0 ^b^	3.13 ± 0.0 ^b^	12.5 ± 0.0 ^a^	–
***C. coli***	6.25 ± 0.0 ^a^	25.0 ± 0.0 ^a^	12.5 ± 0.0 ^a^	12.5 ± 0.0 ^a^	–	6.25 ± 0.0 ^a^

Values are mean ± SD (*n* = 3). Different superscript letters within each column indicate significant differences (*p* < 0.05; one-way ANOVA, Tukey’s test). “–”: no activity detected.

**Table 4:** Effect of NaCl concentration on *C. jejuni* growth (%) in the presence of EOs of *M. communis, M. communis,* and *E. globulus* at their MIC levels.

NaCl (%)	Control	*Essential oils*		
		*M. communis*	*T. algeriensis*	*E. globulus*
**0.0**	100.0 ± 0.0 ^a^	66.4 ± 1.3 ^b^	67.4 ± 0.8 ^b^	1.0 ± 0.2 ^c^
**2.5**	91.2 ± 0.6 ^a^	58.4 ± 0.9 ^b^	66.4 ± 1.1 ^b^	0.8 ± 0.1 ^c^

Values represent mean ± SD (*n* = 3). Different letters within the same row indicate significant differences (*p* < 0.05; two-way ANOVA, Tukey’s test). No growth was observed at 5% and 10% NaCl for any sample, so these values are not reported.

## Data Availability

The original contributions presented in the study are included in the article. Further inquiries can be directed to the corresponding author.

## Abbreviations

The following abbreviations are used in this manuscript:

MIC	Minimum Inhibitory Concentration
MBC	Minimum Bactericidal Concentration
EOs	Essential Oils
